# Trend and Risk Factors of Diverticulosis in Japan: Age, Gender, and Lifestyle/Metabolic-Related Factors May Cooperatively Affect on the Colorectal Diverticula Formation

**DOI:** 10.1371/journal.pone.0123688

**Published:** 2015-04-10

**Authors:** Nobutake Yamamichi, Takeshi Shimamoto, Yu Takahashi, Yoshiki Sakaguchi, Hikaru Kakimoto, Rie Matsuda, Yosuke Kataoka, Itaru Saito, Yosuke Tsuji, Seiichi Yakabi, Chihiro Takeuchi, Chihiro Minatsuki, Keiko Niimi, Itsuko Asada-Hirayama, Chiemi Nakayama, Satoshi Ono, Shinya Kodashima, Daisuke Yamaguchi, Mitsuhiro Fujishiro, Yutaka Yamaji, Ryoichi Wada, Toru Mitsushima, Kazuhiko Koike

**Affiliations:** 1 Department of Gastroenterology, Graduate School of Medicine, The University of Tokyo, 7-3-1, Hongo, Bunkyo-ku, Tokyo, Japan; 2 Kameda Medical Center Makuhari, CD-2, 1–3, Nakase, Mihama-ku, Chiba-city, Japan; University Hospital Llandough, UNITED KINGDOM

## Abstract

**Background:**

Despite the marked increase of diverticulosis, its risk factors have not been adequately elucidated. We therefore aim to identify significantly associated factors with diverticulosis. We also aim to investigate the present state of diverticulosis in Japan.

**Methods:**

We reviewed the medical records from 1990 to 2010 that included the data of consecutive 62,503 asymptomatic colonoscopy examinees from the general population in Japan. Most recent 3,327 examinees were analyzed with 16 background factors.

**Results:**

Among the 62,503 subjects (47,325 men and 15,178 women; 52.1 ± 9.2 years old), diverticulosis was detected in 11,771 subjects (18.8%; 10,023 men and 1,748 women). The incidences of diverticulosis in 1990-2000 and 2001-2010 were respectively 13.0% (3,771 of 29,071) and 23.9% (8,000 of 33,432): the latter was much higher than the former in all age groups and for both genders. Considering the anatomical locations of colorectal diverticula, left-sided ones have markedly increased with age but not significantly changed with times. Univariate analyses of the 3,327 subjects showed significant association of diverticulosis with four basic factors (age, sex, body mass index, blood pressure), three life style-related factor (smoking, drinking, severe weight increase in adulthood), and two blood test values (triglyceride, HbA1c). The multiple logistic analysis calculating standardized coefficients (β) and odds ratio (OR) demonstrated that age (β = 0.217-0.674, OR = 1.24-1.96), male gender (β = 0.185, OR = 1.20), smoking (β = 0.142-0.200, OR = 1.15-1.22), severe weight increase in adulthood (β = 0.153, OR = 1.17), HbA1c (β = 0.136, OR = 1.15), drinking (β = 0.109, OR = 1.11), and serum triglyceride (β = 0.098, OR = 1.10) showed significantly positive association with diverticulosis whereas body mass index and blood pressure did not.

**Conclusions:**

The large-scale data of asymptomatic colonoscopy examinees from the general population from 1990 to 2010 indicated that the prevalence of diverticulosis is still increasing in Japan. Age, male gender, smoking, severe weight increase in adulthood, serum HbA1c, drinking, and serum triglyceride showed significant positive association with diverticulosis.

## Introduction

The exact prevalence of diverticulosis is difficult to define, because most subjects with diverticulosis might be asymptomatic and also because the large intestine of healthy individuals is not usually examined [1]. Nevertheless, it is broadly accepted that rates of diverticulosis have obviously increased over times worldwide [1–5]. In Japan, several early studies reported the prevalence of diverticulosis based on the barium enema examination: 1.6% in 1975 [6], 7.8% in 1983 [7], 13.3% in 1987 [8], 15.7% in 1995 [9], and 23.6% in 2000 [10]. These reports indicated that prevalence of Japan had increased like Western countries [2, 3]. However, recent reports evaluating the prevalence of diverticulosis among the asymptomatic subjects from the general population are few: thence, one of the aims of this study is investigating the present state of diverticulosis in Japan, one of the typical developing countries in East Asia.

Despite the marked increase of disease rate in the last several decades [1], pathogenesis and risk factors of diverticulosis have not been adequately elucidated [4]. For the basic factors, advancing age is the definite risk factor of diverticulosis validated by many previous studies [6, 7, 9, 11–13]. On the contrary, an association of gender with diverticulosis is still controversial [6, 14, 15]. Low-fiber diet has been repeatedly reported as the risk factor of diverticulosis [11, 16–18], but a recent study achieved the opposite result [13]. As for drinking, results of some reports considered alcohol as a risk factor of diverticulosis whereas other reports did not [19–22]. Association of smoking with diverticulosis is also controversial [13, 21–23]. Recently, several studies indicated that body mass index and/or obesity may be associated with diverticulosis [24–26]. As a whole, definitively associated factors for diverticulosis have not been identified except for advancing age. Therefore, the main purpose of this study is to evaluate associations of several background factors with diverticulosis.

## Methods

### Asymptomatic colonoscopy examinees from the general Japanese population for 21 years

**Fig 1:**
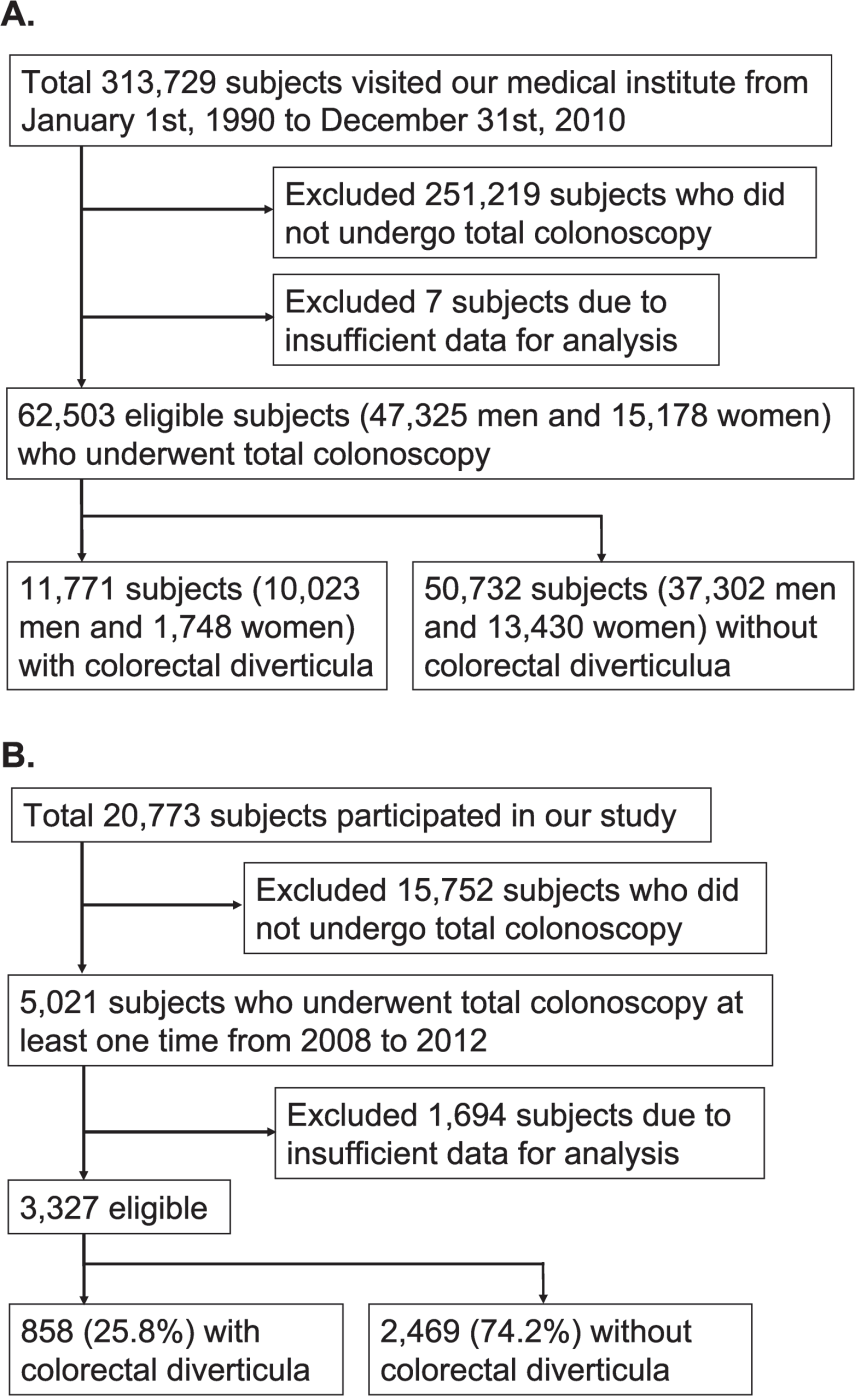
Two flow charts for the present study. (A) Flowchart to select the general asymptomatic colonoscopy examinees for an epidemiologic survey of 21 years in Japan. (B) Flowchart for the selection of study subjects to evaluate univariate and multivariate association between diverticulosis and several background factors.

Of the total 313,729 asymptomatic general adults who visited our medical institute (Kameda Medical Center Makuhari, Chiba, Japan) from 1990 to 2010, we retrospectively reviewed the medical records of consecutive 62,503 subjects (47,325 men and 15,178 women) who underwent total colonoscopy as part of the medical checkup (Fig 1A).

### Study subjects to evaluate the associated background factors of diverticulosis

Of the 20,773 subjects who received medical checkup at our medical institute and approved participating in our study, 5,021 subjects underwent colonoscopy from 2008 to 2012 (Fig 1B). This study was approved by the ethics committee of the University of Tokyo, and written informed consents were obtained from all the study participants according to the Declaration of Helsinki.

### Total colonoscopy and diagnosis of diverticulosis

For bowel preparation for colonoscopy, each subject ingested 2 liters of polyethylene glycol electrolyte (PEG) solution. In case of inadequate preparation, the examinee additionally ingested 0.5–1 litters of PEG solution. After confirming that the bowel's output runs clear, total colonoscopy was performed by well-trained endoscopists both in the morning and afternoon. In case of failure to reach the cecum, the subject was omitted in the present analysis. During the procedure of total colonoscopy, the locations of diverticula were documented. The anatomical locations of colorectal diverticula were classified into cecum, ascending colon, transverse colon, descending colon, sigmoid colon, and rectum.

### Categorization of blood pressure

According to the guideline of the Japanese society of hypertension [27], total subjects were classified into three classes as follows; i) optimal blood pressure: systolic blood pressure (SBP) lower than 120 mmHg and diastolic blood pressure (DBP) lower than 80 mmHg, ii) hypertension: SBP equal or more than 140 mmHg or DBP equal or more than 90 mmHg, and iii) normal range blood pressure: a combination of SBP and DBP not fallen under optimal blood pressure and hypertension.

### Evaluation of serum anti-*Helicobacter pylori* IgG, Alcohol Drinking, and Smoking

Serum anti-*Helicobacter pylori* IgG was measured using a commercial kit (E-plate “EIKEN” Helicobacter pylori antibody, Eiken Chemical Co LTD., Tokyo, Japan) as we previously reported [28–30]. According to the manufacture’s instruction, the titer of anti-*Helicobacter pylori* IgG ≥10 U/ml was considered as *Helicobacter pylori*-positive.

For alcohol drinking, the study subjects were scored based on the 5-grade scale: never (0/month), seldom (1-2/month), sometimes (1-2/week), often (3-5/week), and always (6-7/week, almost every day) [28, 30]. These further classified into “rarely drinking” (never or seldom) or “usually drinking” (sometimes, often, or always) [28, 29, 31]. For smoking, the subjects were classified into three groups: “current smoker”, “past habitual smoker”, and “lifelong nonsmoker” [30].

Based on our previous study [32, 33], we used additional four questions to assess the lifestyle-related factors: 1) Has your body weight markedly increased in adulthood (more than 10 kg from age 20 years)? 2) Do you have a feeling of inadequate sleep? 3) Do you have a habit of frequent skipping of breakfast (more than three times a week)? 4) Do you have a habit of having dinner within two hours before going to bed?

### Statistical Analyses

We used JMP 10 software or SAS 9.1.3 (SAS Institute Inc. Cray, NC, USA) for statistical analyses. In the univariate analyses, categorical data were analyzed by Pearson’s chi-square test, and continuous data were analyzed by Welch’s t-test. Covariates associated with the presence of diverticulosis were carefully selected based on known confounders and clinical knowledge as follows: age (categorical data), sex (categorical data), body mass index (categorical data), blood pressure (categorical data), habit of smoking (categorical data), habit of drinking (categorical data), severe weight increase in adulthood (categorical data), feeling of inadequate sleep (categorical data), habit of frequent skipping of breakfast (categorical data), habit of having dinner within two hours before going to bed (categorical data), anti-*Helicobacter pylori* IgG (categorical data), serum T-chol (total cholesterol, continuous data, [mg/dl]), serum LDL-chol (low-density lipoprotein cholesterol, continuous data, [mg/dl]), serum triglyceride (continuous data, [mg/dl]), serum albumin (continuous data, [g/dl]), and serum HbA1c (glycated hemoglobin, continuous data, [%]).

To compare the incidence trend of anatomical locations of colorectal diverticula in 1990–2000 and that in 2001–2010, we constructed the interaction terms of diverticula’s locations with advancing age or an examination period to apply “Zero-Inflated Poisson model” [34]. To evaluate relative strengths of predictors on diverticulosis, they were calculated by fitting a logistic regression model using all the relevant variables, which were derived from the univarite analyses. In all the analyses, *p* values <0.05 were considered as statistically significant.

## Results

### Prevalence of diverticulosis has been still increasing in Japan

The total 62,503 asymptomatic colonoscopy examinees (mean age 52.1 ± 9.2 years; range 20 to 93 years) from 1990 to 2010 were analyzed. Colorectal diverticula were detected in 18.8% of them (11,771 subjects, Fig 1A), which comprised 10,023 men (21.2% of 47,325 men) and 1,748 women (11.5% of 15,178 women). We divided them into 29,071 subjects in the first half 11 years (1990–2000) and 33,432 subjects in the second half 10 years (2001–2010). Regardless of genders or examination periods, incidence of diverticulosis increases with age (Table 1), which is consistent with previous many reports [1, 6, 7, 9, 11–13, 35].

**Table 1:** Prevalence of diverticulosis in the six age groups of the 29,071 colonoscopy examinees from 1990 to 2000 and 33,432 colonoscopy examinees from 2001 and 2010 in Japan.

Age groups	Prevalence of diverticulosis	Number of the total examinees	Prevalence of diverticulosis in the male examinees	Number of the male examinees	Prevalence of diverticulosis in the female examinees	Number of the female examinees
1990–2000						
<30	2 (1.4%)	142	2 (2.0%)	101	0 (0%)	41
≥30 and <40	176 (6.3%)	3,094	154 (6.1%)	2,526	22 (3.9%)	568
≥40 and <50	1,290 (10.8%)	11,969	1,131 (12.6%)	8,981	159 (5.3%)	2,988
≥50 and <60	1,511 (15.2%)	9,913	1,272 (17.7%)	7,177	239 (8.7%)	2,736
≥60 and <70	647 (19.5%)	3,325	524 (21.6%)	2,425	123 (13.7%)	900
≥70	145 (23.1%)	628	104 (23.9%)	436	41 (21.4%)	192
Total	3,771 (13.0%)	29,071	3,187 (14.7%)	21,646	584 (7.9%)	7,425
2001–2010						
<30	4 (5.7%)	70	3 (6.5%)	46	1 (4.2%)	24
≥30 and <40	195 (10.8%)	1,798	166 (11.8%)	1,408	29 (7.4%)	390
≥40 and <50	1,419 (18.4%)	7,721	1,273 (20.9%)	6,097	146 (9.0%)	1,624
≥50 and <60	3,960 (25.9%)	15,312	3,441 (29.1%)	11,807	519 (14.8%)	3,505
≥60 and <70	1,988 (28.7%)	6,938	1,614 (31.3%)	5,160	374 (21.0%)	1,778
≥70	434 (27.2%)	1,593	339 (29.2%)	1,161	95 (22.0%)	432
Total	8,000 (23.9%)	33,432	6,836 (26.6%)	25,679	1,164 (15.0%)	7,753

**Fig 2:**
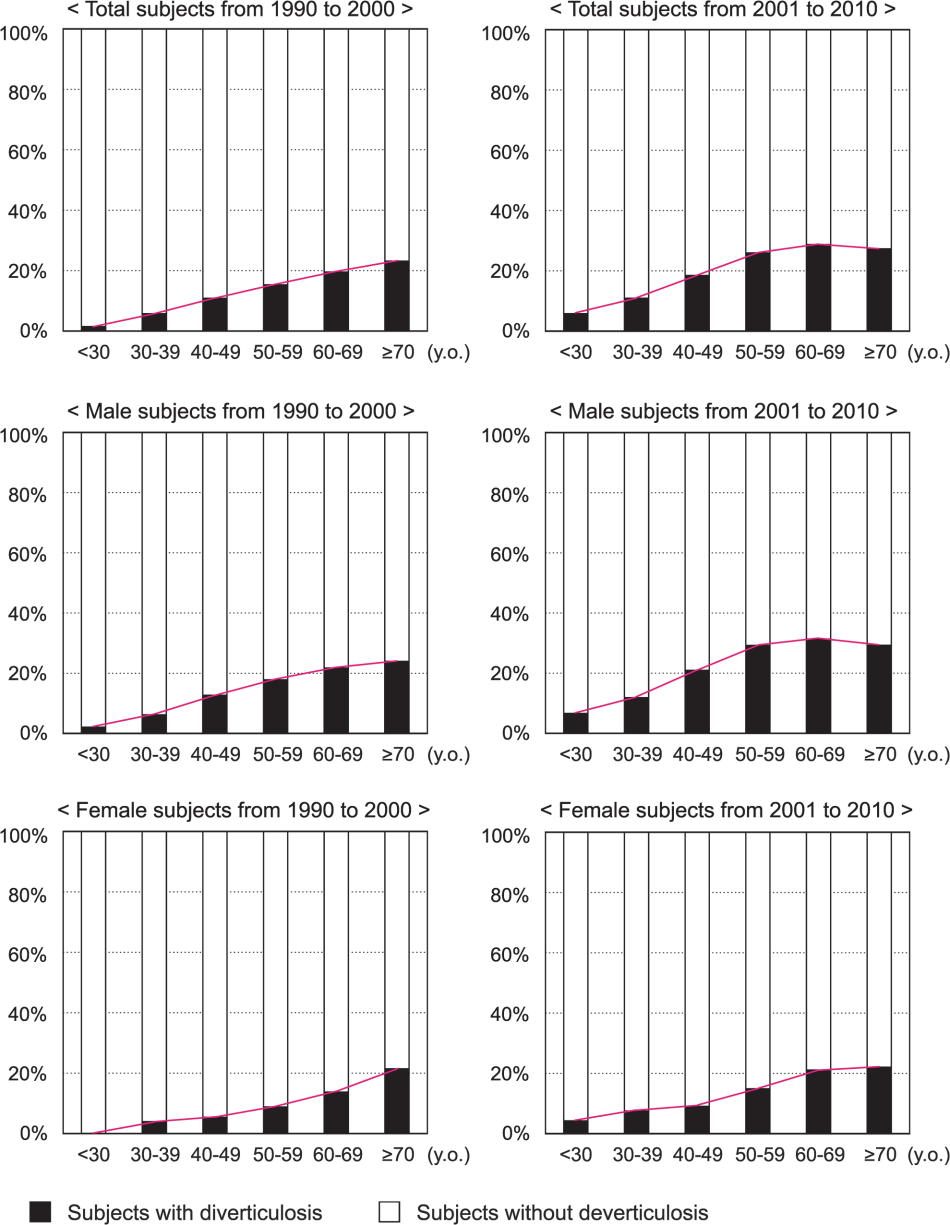
Prevalence of diverticulosis in Japan from 1990 to 2000 and from 2001 to 2010.

As shown in Table 1 and Fig 2, the disease rates of diverticulosis in 2001–2010 were higher than those in 1990–2000 for both genders and in all the age groups. As described in Introduction, the prevalence of diverticulosis in Japan had increased from a few percents in 1970s to about 20% around 2000 on the basis of barium enema studies [6–10]. Our results indicated that the prevalence of diverticulosis has been still increasing in Japan, although it has not reached the still higher disease rates of diverticulosis in Western countries such as 39.9% (318 of 796) in France [12] or 55.5% (497 of 896) in the United States [35].

### Left-sided colorectal diverticula increase markedly with age but not significantly with times

**Fig 3:**
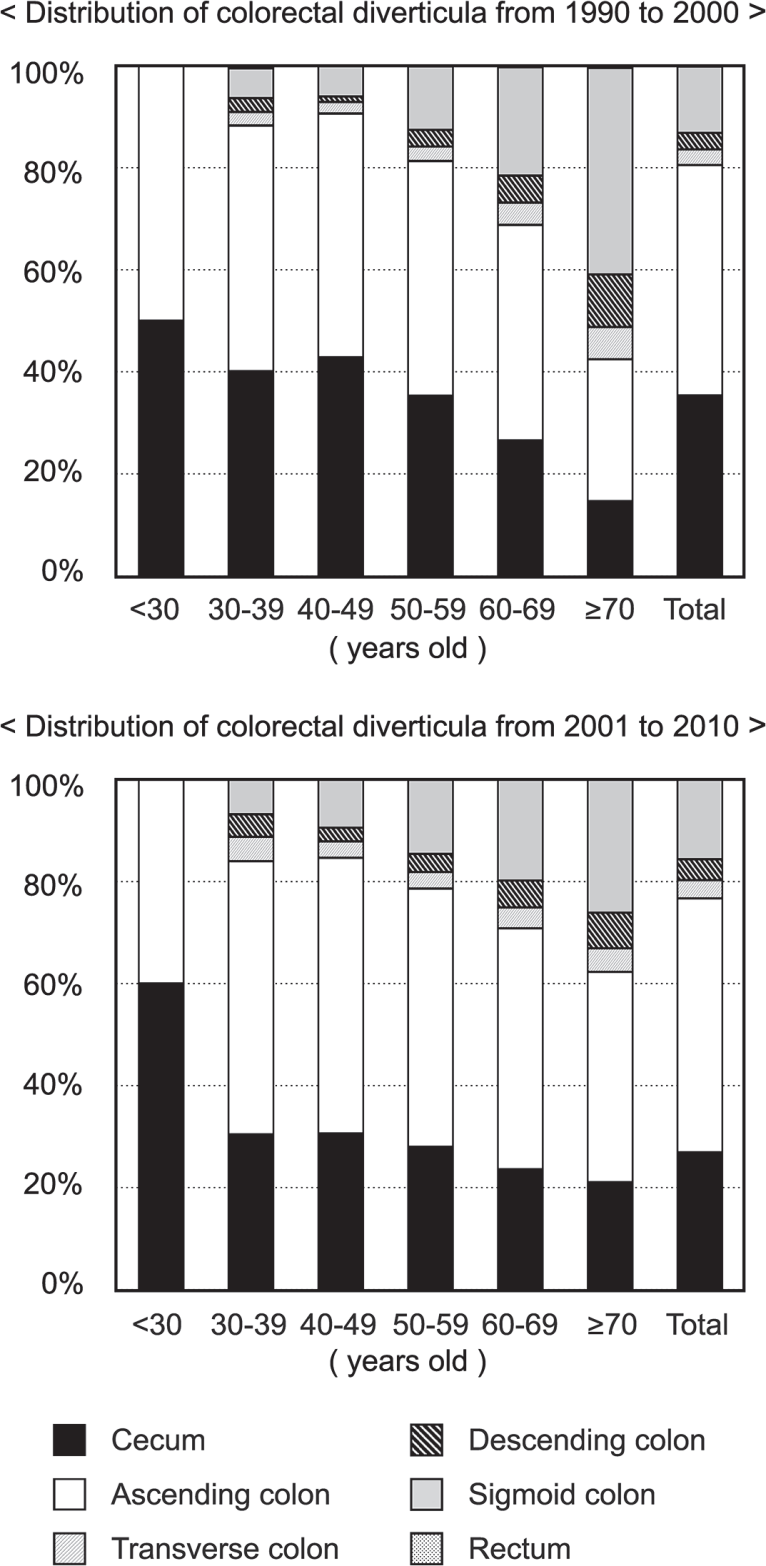
Anatomical location of colorectal diverticula in the first half 11 years (from 1990 to 2000) and the second half 10 years (from 2001 to 2010). The disease rates of diverticula in cecum, ascending colon, transverse colon, descending colon, sigmoid colon, and rectum in the six age groups are shown as cumulative bar chart.

Several previous studies reported that the incidence of diverticula in the left-sided colon increases with advancing age [7, 9, 10]. Our results based on the data of colonoscopy examinees over 21 years similarly showed that the rates of diverticula in sigmoid and descending colon obviously increase with age, and those of diverticula in cecum and ascending colon apparently reduce in parallel (Fig 3, Table 2, S1 Table and S2 Table).

**Table 2:** Anatomical locations of colorectal diverticula in the six age groups of the 29,071 colonoscopy examinees from 1990 to 2000 and 33,432 colonoscopy examinees from 2001 and 2010 in Japan.

Age groups	Cecum	Ascending Colon	Transverse Colon	Descending Colon	Sigmoid Colon	Rectum	Number of examinees in each age group
1990–2000							
<30	1 (0.70%)	1 (0.70%)	0 (0.0%)	0 (0.0%)	0 (0.0%)	0 (0.0%)	142
≥30 and <40	75 (2.4%)	90 (2.9%)	5 (0.16%)	5 (0.16%)	11 (0.36%)	1 (0.03%)	3,094
≥40 and <50	643 (5.4%)	718 (6.0%)	34 (0.28%)	16 (0.13%)	90 (0.75%)	1 (0.01%)	11,969
≥50 and <60	696 (7.0%)	907 (9.1%)	56 (0.56%)	64 (0.65%)	246 (2.5%)	3 (0.03%)	9,913
≥60 and <70	236 (7.1%)	376 (11.3%)	39 (1.2%)	47 (1.4%)	189 (5.7%)	3 (0.09%)	3,325
≥70	30 (4.8%)	57 (9.1%)	13 (2.1%)	21 (3.3%)	83 (13.2%)	1 (0.16%)	628
Total	1,681 (5.8%)	2,149 (7.4%)	147 (0.51%)	153 (0.53%)	619 (2.1%)	9 (0.03%)	29,071
2001–2010							
<30	3 (4.3%)	2 (2.9%)	0 (0.0%)	0 (0.0%)	0 (0.0%)	0 (0.0%)	70
≥30 and <40	76 (4.2%)	134 (7.5%)	12 (0.67%)	11 (0.61%)	17 (0.95%)	0 (0.0%)	1,798
≥40 and <50	550 (7.1%)	973 (12.6%)	58 (0.75%)	48 (0.62%)	170 (2.2%)	0 (0.0%)	7,721
≥50 and <60	1,576 (10.3%)	2,852 (18.6%)	183 (1.2%)	201 (1.3%)	817 (5.3%)	3 (0.02%)	15,312
≥60 and <70	710 (10.2%)	1,425 (20.5%)	124 (1.8%)	158 (2.3%)	596 (8.6%)	1 (0.01%)	6,938
≥70	136 (8.5%)	267 (16.8%)	30 (1.9%)	45 (2.8%)	169 (10.6%)	0 (0.0%)	1,593
Total	3,051 (9.1%)	5,653 (16.9%)	407 (1.2%)	463 (1.4%)	1,769 (5.3%)	4 (0.01%)	33,432

On the contrary, distributions of colorectal diverticula in the first half 11 years and in the second half 10 years are rather similar (Fig 3). By applying the “Zero-Inflated Poisson model”, we compared the incidence trend of the anatomical locations of colorectal diverticula in 1990–2000 with that in 2001–2010. This statistical evaluation revealed that there is no significant difference in the location of colorectal diverticula between the two periods (*p* = 0.4042).

### Significantly associated factors of diverticulosis

From the 5,021 general asymptomatic colonoscopy examinees who approved participating in our study, 1,694 subjects were excluded due to insufficient data for analyses (Fig 1B). Of the 3,327 eligible subjects (2,485 men and 842 women; mean age 55.0 ± 9.1 years; range 20 to 86 years), colorectal diverticula were detected in 858 subjects (25.8%, Table 3), which comprised 734 men (29.5% of 2,485 men) and 124 women (14.7% of 842 women). To identify predictive factors of diverticulosis, we statistically evaluated association of diverticulosis with four basic factors (age, sex, body mass index, and blood pressure), six lifestyle-related factors (including smoking and drinking), and six blood test values (Table 3).

**Table 3:** Univariate analyses evaluating associations between diverticulosis and the 16 background factors using the data of 3,327 study subjects.

Factor	858 subjects with diverticulosis	2,469 subjects without diverticulosis			*p* value
Age (years old)					<0.0001 *
<40	15 (8.5%)	161 (91.5%)			
≥40 and <50	109 (16.1%)	568 (83.9%)			
≥50 and <60	403 (27.8%)	1,049 (72.2%)			
≥60 and <70	277 (32.5%)	574 (67.5%)			
≥70	54 (31.6%)	117 (68.4%)			
Sex					<0.0001 *
Female	124 (14.7%)	718 (85.3%)			
Male	734 (29.5%)	1,751 (70.5%)			
Body mass index					<0.0001 *
≥18.5 and <25	531 (23.8%)	1,697 (76.2%)			
<18.5	17 (11.0%)	138 (89.0%)			
≥25	310 (32.8%)	634 (67.2%)			
Blood pressure					<0.0001 *
Optimal blood pressure	592 (23.9%)	1,887 (76.1%)			
Normal range blood pressure	145 (31.5%)	316 (68.5%)			
Hypertension	121 (31.3%)	266 (68.7%)			
Smoking					<0.0001 *
Lifelong nonsmoker	259 (18.6%)	1,134 (81.4%)			
Past habitual smoker	372 (30.4%)	850 (69.6%)			
Current smoker	227 (31.9%)	485 (68.1%)			
Alcohol drinking					<0.0001 *
Rarely drinking	229 (20.4%)	892 (79.6%)			
Usually drinking	629 (28.5%)	1,577 (71.5%)			
Severe weight increase in adulthood (more than 10 kg from age 20 years)					<0.0001 *
No	383 (20.8%)	1,459 (79.2%)			
Yes	475 (32.0%)	1,010 (68.0%)			
Feeling of inadequate sleep					0.9475
No	303 (25.7%)	875 (74.3%)			
Yes	555 (25.8%)	1,594 (74.2%)			
Habit of frequent skipping of breakfast (more than three times a week)					0.5719
No	761 (25.9%)	2,172 (74.1%)			
Yes	97 (24.6%)	297 (75.4%)			
Habit of having dinner within two hours before going to bed					0.5206
No	620 (25.5%)	1,812 (74.5%)			
Yes	238 (26.6%)	657 (73.4%)			
Anti-*Helicobacter pylori* IgG					0.0642
Negative	472 (24.7%)	1,436 (75.3%)			
Positive	297 (27.8%)	770 (72.2%)			
Serum T-chol (mg/dl)	203.5 ± 30.8	203.8 ± 31.5			0.8565
Serum LDL-chol (mg/dl)	126.8 ± 28.7	125.3 ± 30.3			0.1845
Serum triglyceride (mg/dl)	132.5 ± 80.4	111.9 ± 70.4			<0.0001 *
Serum albumin (g/dl)	4.35 ± 0.23	4.36 ± 0.24			0.4147
Serum HbA1c (%)	5.69 ± 0.80	5.50 ± 0.57			<0.0001 *

T-chol, total cholesterol; LDL-chol, low-density lipoprotein cholesterol; HbA1c, hemoglobin A1c. The level of significance in each factor was set at *p*<0.05 (*).

Of the 16 analyzed variables, univariate analyses showed the statistically significant association between diverticulosis and all the four basic factors: namely, older age, male gender, higher body mass index, and hypertension were significantly associated with the presence of colorectal diverticula (Table 3). In addition, three lifestyle-related factors (smoking, alcohol drinking, and severe weight increase in adulthood) and two blood test values (serum triglyceride and HbA1c) denoted strong association with diverticulosis (Table 3).

Based on the univariate analyses, we further performed the multivariate logistic analysis evaluating associations of diverticulosis with selected nine variables (Table 4). Judging from the *p* values and standardized coefficients, diverticulosis showed significantly positive association with older age, male gender, smoking, severe weight increase in adulthood, serum HbA1c value, alcohol drinking, and serum triglyceride value in this order (Table 4). On the contrary, body mass index and blood pressure did not denote meaningful association with diverticulosis.

**Table 4:** Multivariate analysis evaluating associations between diverticulosis and the selected nine background factors using the data of 3,327 study subjects.

Factor	Standardized coefficients	Odds ratio (95% C.I.)	*p* value
Age (years old)			<0.0001 *
<40	reference	reference	reference
≥40 and <50	0.217	1.24 (1.00–1.59)	0.0675
≥50 and <60	0.590	1.80 (1.39–2.42)	<0.0001 *
≥60 and <70	0.674	1.96 (1.56–2.55)	<0.0001 *
≥70	0.342	1.41 (1.23–1.63)	<0.0001 *
Sex (male)	0.185	1.20 (1.08–1.35)	0.0011 *
Smoking			
Lifelong nonsmoker	reference	reference	reference
Past habitual smoker	0.142	1.15 (1.04–1.28)	0.0055 *
Current smoker	0.200	1.22 (1.11–1.35)	<0.0001 *
Severe weight increase in adulthood (more than 10 kg from age 20 years)	0.153	1.17 (1.06–1.28)	0.0011 *
Serum HbA1c	0.136	1.15 (1.06–1.24)	0.0006 *
Alcohol drinking	0.109	1.11 (1.02–1.22)	0.0199 *
Serum triglyceride	0.098	1.10 (1.02–1.20)	0.0182 *
Body mass index			
≥18.5 and <25	reference	reference	reference
<18.5	-0.104	0.90 (0.80–1.00)	0.0706
≥25	0.072	1.07 (0.98–1.18)	0.1152
Blood pressure			
Optimal blood pressure	reference	reference	reference
Normal range blood pressure	0.064	1.07 (0.98–1.15)	0.1122
Hypertension	0.044	1.05 (0.97–1.13)	0.2694

C.I., confidence interval; HbA1c, hemoglobin A1c. The level of significance in each factor was set at *p*<0.05 (*).

## Discussion

The present analysis of the 3,327 colonoscopy examinees from 2008 to 2012 showed that incidence of diverticulosis is 25.8% (858 subjects, Fig 1B). Past studies focusing on diverticulosis in Japan were mostly based on the barium enema examination, in which the prevalence of colorectal diverticula has been reported to increase from 1.6% to 23.6% in the quarter of century [6–10]. More recently, two studies analyzing the data from colonoscopy examinees were reported, in which the incidence of diverticulosis was 24.5% (165 of 672 subjects) in 2008 [15] or 25.0% (542 of 2164 subjects) in 2013 [22]. These two studies evaluated the patients who consulted to a doctor for some physical problems, whereas our study subjects were asymptomatic general adults who underwent total colonoscopy for medical checkup. Nevertheless, the disease rates in the three studies were quite similar: at present, the prevalence of diverticulosis would be about 25% in Japan. Recently, Niikura *et al* compared the diagnostic ability of barium enema and colonoscopy for diverticulosis: they reported that only half of diverticula were detected by colonoscopy compared with barium enema [36]. Niikura’s study suggested that present true prevalence of diverticulosis in Japan may be even higher than 25%, which is not contradictory to the above-mentioned colonoscopy-based studies including the present one [15, 22].

Of the 16 background factors we examined, the univarite and multivariate analyses demonstrated that diverticulosis has significantly positive associations with two basic factors (age and male gender), two blood test values (serum triglyceride and HbA1c), and three life style-related factors (smoking, alcohol drinking, and severe weight increase in adulthood). Judging from the values of standardized coefficients and odds ratios (Table 4), we could not identify the overwhelmingly associated factors for diverticulosis. These results suggest that multifactorial mechanisms should work on the formation of colorectal diverticula.

Consistent with previous many reports [1, 6, 7, 9, 11–13, 35], advancing age shows the strongest association with diverticulosis. Long-lasting increased prevalence of diverticulosis [1–10] may be partly due to advent of aging society in Japan. However, disease rates of diverticulosis in 2001–2010 were much higher than those in 1990–2000, even when the subjects were in the same age groups (Table 1): this indicates that other crucial factors must have great influences on the marked increase of diverticulosis.

Our results also revealed positive associations of diverticulosis with smoking and drinking, both of which were controversial in the previous studies [13, 19–21, 23]. In Japan, the number of smokers has been obviously decreased in the recent 50 years, which is opposite to the increasing incidence of diverticulosis (Fig 2). This suggests that other risk and/or preventive factors have overcome the possible influence of decreasing rate of smoking in Japan. On the other hand, alcohol consumption has gradually increased in Japan since the end of World War II (1945) [37, 38], which may be one of the important causes of remarkably increased diverticulosis.

When thought of a noticeable increase of diverticulosis in the recent several decades [1, 2], we speculate that various metabolic-related factors should have considerable roles on the formation of diverticula. Though the *p* value was not significant, higher body mass index tends to accompany with higher standardized coefficient and higher odds ratio of diverticulosis (Table 4). Among our identified significant variables, severe weight increase in adulthood, serum HbA1c, and serum triglyceride are metabolic-related factors, which may work on the increase of colorectal diverticula. Among them, serum HbA1c is one of the most important markers for diabetes mellitus, the prevalence of which has regrettably increased in the recent 30 years in Japan [39–41]. We speculate the increased prevalence of diabetes not only in Japan but worldwide [42, 43] may be a great risk of diverticulosis in the past, present, and future.

There are some limitations in the present study. First, this is the single center study with cross-sectional or retrospective design: a future prospective study is necessary to confirm the results of the present study. Second, detectability of the colorectal diverticula may change during the 21 years (between 1990 and 2010). As preparation and procedure of total colonoscopy have not been changed in the 21 years, and also as the skill level of the endoscopists has been kept high in our institute, we think the detectability of colorectal diverticula has not been altered in the 21 consecutive years. Nevertheless, we cannot completely deny the possibility that improved colonoscopes over 20 years may increase the detection rate of diverticula. Third, we did not have detailed information for smoking and drinking. Data of quantified alcohol intake or duration of smoking might improve our analyses. However, we have already used the 5-grade scales of alcohol intake and 3-group classification of smoking in our six previous studies [28–33]: therefore, we think our categorization for drinking and smoking has been validated to a certain extent. Fourth, we had no available information about the diet and physical activity of the study subjects. In particular, it was a matter of regret that we had no data about low/high fiber diet, influence of those upon diverticulosis is still controversial [11, 13, 16–18].

## Conclusions

The large-scale study based on the 62,503 asymptomatic colonoscopy examinees from the general population for 21 years showed that the prevalence of colorectal diverticulosis has been increasing up to about 25% in Japan. Multivariate logistic analysis further demonstrated that the presence of colorectal diverticula has significantly positive association with two basic factors (age and male gender) and five lifestyle/metabolic-related factors (smoking, alcohol drinking, serum triglyceride, serum HbA1c, and severe weight increase in adulthood).

## Supporting Information

S1 Table Anatomical locations of colorectal diverticula in the six age groups of the 21,646 male colonoscopy examinees from 1990 to 2000 and 25,679 male colonoscopy examinees from 2001 and 2010 in Japan. (DOC)

S2 Table Anatomical locations of colorectal diverticula in the six age groups of the 7,425 female colonoscopy examinees from 1990 to 2000 and 7,753 female colonoscopy examinees from 2001 and 2010 in Japan. (DOC)
