# Supplementary material for: Trend and Risk Factors of Diverticulosis in Japan: Age, Gender, and Lifestyle/Metabolic-Related Factors May Cooperatively Affect on the Colorectal Diverticula Formation
Source: PLoS One. 2015 Apr 10;10(4):e0123688. doi: 10.1371/journal.pone.0123688 (PMC4393308; doi:10.1371/journal.pone.0123688)
Supplement: S1 Table — (DOC) [file pone.0123688.s001.doc]

**Table S1.** Anatomical locations of colorectal diverticula in the six age groups of the 21,646 male colonoscopy examinees from 1990 to 2000 and 25,679 male colonoscopy examinees from 2001 and 2010 in Japan.

| **Age groups** | **Cecum** | **Ascending Colon** | **Transverse Colon** | **Descending Colon** | **Sigmoid Colon** | **Rectum** | **Number of male examinees in each age group** |
| --- | --- | --- | --- | --- | --- | --- | --- |
| 1990-2000 |  |  |  |  |  |  |  |
| <30 | 1 (0.99%) | 1 (0.99%) | 0 (0.0%) | 0 (0.0%) | 0 (0.0%) | 0 (0.0%) | 101 |
| ≥30 and <40 | 66 (2.6%) | 80 (3.2%) | 5 (0.20%) | 5 (0.20%) | 11 (0.44%) | 1 (0.04%) | 2,526 |
| ≥40 and <50 | 587 (6.5%) | 635 (7.1%) | 28 (0.31%) | 15 (0.17%) | 66 (0.73%) | 0 (0.0%) | 8,981 |
| ≥50 and <60 | 613 (8.5%) | 788 (11.0%) | 48 (0.67%) | 57 (0.79%) | 186 (2.6%) | 1 (0.04%) | 7,177 |
| ≥60 and <70 | 201 (8.3%) | 318 (13.1%) | 34 (1.4%) | 43 (1.8%) | 152 (6.3%) | 3 (0.12%) | 2,425 |
| ≥70 | 22 (5.0%) | 48 (11.0%) | 10 (2.3%) | 12 (2.8%) | 53 (12.2%) | 0 (0.0%) | 436 |
| Total | 1,490 (6.9%) | 1,870 (8.6%) | 125 (0.58%) | 132 (0.61%) | 468 (2.2%) | 7 (0.03%) | 21,646 |
| 2001-2010 |  |  |  |  |  |  |  |
| <30 | 3 (4.3%) | 2 (2.9%) | 0 (0.0%) | 0 (0.0%) | 0 (0.0%) | 0 (0.0%) | 46 |
| ≥30 and <40 | 76 (4.2%) | 134 (7.5%) | 12 (0.67%) | 11 (0.61%) | 17 (0.95%) | 0 (0.0%) | 1,408 |
| ≥40 and <50 | 550 (7.1%) | 973 (12.6%) | 58 (0.75%) | 48 (0.62%) | 170 (2.2%) | 0 (0.0%) | 6,097 |
| ≥50 and <60 | 1,576 (10.3%) | 2,852 (18.6%) | 183 (1.2%) | 201 (1.3%) | 817 (5.3%) | 3 (0.02%) | 11,807 |
| ≥60 and <70 | 710 (10.2%) | 1,425 (20.5%) | 124 (1.8%) | 158 (2.3%) | 596 (8.6%) | 1 (0.01%) | 5,160 |
| ≥70 | 136 (8.5%) | 267 (16.8%) | 30 (1.9%) | 45 (2.8%) | 169 (10.6%) | 0 (0.0%) | 1,161 |
| Total | 3,051 (9.1%) | 5,653 (16.9%) | 407 (1.2%) | 463 (1.4%) | 1,769 (5.3%) | 4 (0.01%) | 25,679 |
